# A successful complete resection for multidrug-resistant giant gastrointestinal stromal tumor invading the transverse colon with multiple liver metastases in a young female: a case report

**DOI:** 10.1186/s40792-024-01947-1

**Published:** 2024-06-14

**Authors:** Kenta Aso, Nobuyuki Takemura, Yuhi Yoshizaki, Fuminori Mihara, Fuyuki Inagaki, Kazuhiko Yamada, Norihiro Kokudo

**Affiliations:** 1 Department of Surgery, National Center for Global Health and Medicine, Hepato-Biliary Pancreatic Surgery Division, 1-21-1 Toyama, Shinjuku-Ku, Tokyo, 162-8655 Japan; 2 Department of Surgery, Upper Abdominal Surgery Division, National Center for Global Health and Medicine, 1-21-1 Toyama, Shinjuku-Ku, Tokyo, 162-8655 Japan; 3 Department of Hepato-Biliary-Pancreatic Surgery and Pediatric Surgery, Saitama Medical Center, Saitama Medical University, 1981 Kamoda,Kawagoe-Shi, Saitama, 350-8550 Japan

**Keywords:** Gastrointestinal stromal tumor, Liver metastasis, Multidrug-resistant, Adolescents, Young adults

## Abstract

**Background:**

Gastrointestinal stromal tumors (GISTs) are rare in young people and are often detected after becoming symptomatic or at an advanced stage. Herein, we report a case of complete reduction surgery for a substantially large malignant gastric GIST with multiple liver metastases in a young woman who successfully resulted in R0 surgery.

**Case presentation:**

An 18-year-old woman presented to our hospital with anorexia and vomiting, and was diagnosed with a 17 cm gastric GIST with transverse colon invasion and multiple liver metastases. Due to being considered unresectable, tyrosine and multi-kinase inhibitor therapy were administered up to the fourth line yielding no response. After careful discussion at a multidisciplinary team conference, pancreatoduodenectomy or distal gastrectomy, transverse colectomy, and resection of the liver metastases were planned. Consequently, distal gastrectomy, transverse colectomy, resection of the liver metastases, and incidental peritoneal metastases were performed. Although the primary goal of the surgery was to reduce the volume of the tumor as much as possible, the results revealed that the complete removal of all detectable tumors was achieved. No recurrence was observed after surgery for 27 months with long-term adjuvant imatinib therapy.

**Conclusions:**

Even for highly advanced GISTs, aggressive surgery followed by adjuvant drug therapy may prolong survival in young patients.

## Background

Gastrointestinal stromal tumors (GISTs) are rare malignancies, accounting for 1–2% of all gastrointestinal neoplasms [1–3]. The median age at diagnosis is 66–69 years [4]. GISTs are rare in the pediatric population and among adolescents and young adults (AYAs); however, when they occur in this demographic, they are often associated with a genetic predisposition [5, 6]. Furthermore, GISTs in adolescents and young adults are often detected after they become symptomatic and are often diagnosed at an advanced stage [7]. However, the clinical features of the GISTs in these patients are not completely understood.

The development of tyrosine kinase inhibitors (TKIs) has significantly improved the management of unresectable and advanced GISTs. However, the therapeutic selection of multidrug-resistant and liver metastatic GISTs is challenging, and clear clinical evidence for surgical resection is insufficient and controversial. Herein, we report the successful resection of a substantially large malignant gastric GIST invading the transverse colon with multiple liver metastases in a young woman.

## Case presentation

**Fig. 1:**
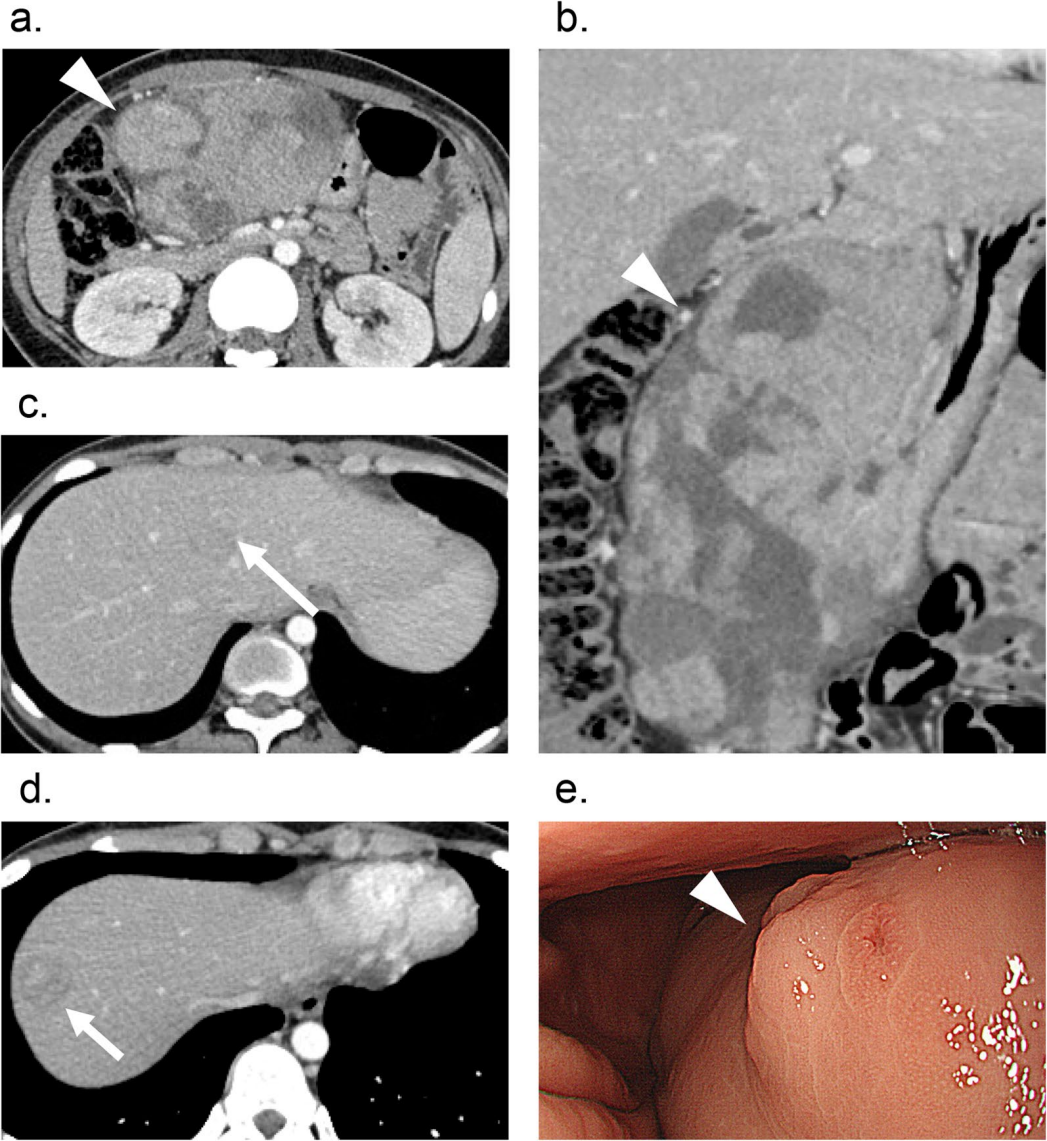
Preoperative imaging findings. **a** Abdominal/pelvic computed tomography (CT) scan with intravenous contrast shows a huge gastric body-enhancing mass, approximately 17 cm in diameter (arrowhead). **b** Coronal view of the CT scan demonstrates a huge heterogeneous hypervascular mass (arrowhead). **c** CT scan showing delayed enhancement of mass lesions in the S4 segment of the liver (large arrow). **d** CT scan showing delayed enhancement of mass lesions in the S8 segment of the liver (small arrow). **e** Gastrointestinal endoscopy detected large submucosal lesion > 10 cm in diameter with multiple ulcers on its surface in the lower body of the stomach (arrowhead)

**Fig. 2:**
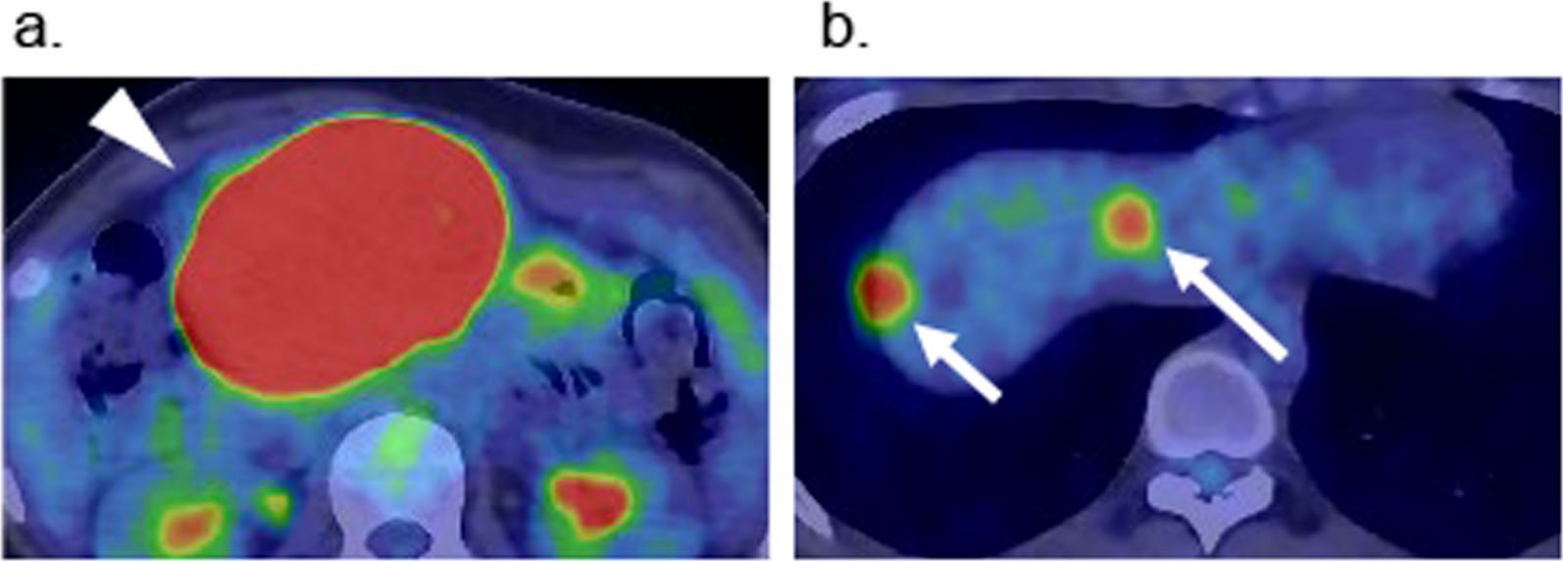
Preoperative FDG-PET/CT imaging. **a** FDG-PET/CT showing strong FDG uptake in the gastric tumor with the SUVmax 37.95 (arrowhead). **b** FDG-PET/CT showing FDG uptake of liver masses located in the S4 segment (SUVmax: 12.3) (large arrow) and S8 segment (SUVmax: 5.0) (small arrow)

An 18-year-old woman presented to our hospital with anorexia and vomiting. Abdominal/pelvic contrast-enhanced computed tomography (CT) revealed a large gastric body mass, approximately 17 cm in diameter (Fig. 1a, b), and delayed enhancement of liver mass lesions in the S4 (Fig. 1c) and S8 segments of the liver (Fig. 1d). Gastrointestinal endoscopy revealed a large submucosal lesion in the lower body of the stomach with ulcers on the surface (Fig. 1e). Biopsy specimens revealed a c-kit-, vimentin-, and CD34-positive spindle-cell GIST. Upon further examination, positron emission tomography with 18F-Fluorodeoxyglucose (FDG) integrated with CT showed strong FDG uptake in the gastric tumor. The maximum standardized uptake value (SUVmax) was 37.95 (Fig. 2a). Furthermore, FDG uptake was observed in the liver masses located in the S4 (SUVmax: 12.3) and S8 (SUVmax: 5.0) segments of the liver (Fig. 2b). Moreover, gadoxetic acid-enhanced dynamic magnetic resonance imaging detected multiple liver metastases < 10 mm in diameter (S1, S4, S5, S7, S6, and S8 segments). Based on these findings, the patient was diagnosed with a giant advanced gastric GIST with multiple liver metastases.

The patient’s treatment strategy was discussed at the multidisciplinary team (MDT) conference by surgical gastroenterologists, oncologists, and radiologists. Initially, the GIST was considered unresectable due to its large size, distant metastases, and the difficulty of achieving R0 resection. Therefore, imatinib therapy was administered as first line treatment, with a daily dosage of 400 mg imatinib mesylate. However, primary tumor growth was confirmed after 8 months of imatinib therapy. Second line treatment with a daily dose of 47.5 mg sunitinib (for 4 weeks with 2 weeks off), third line treatment with TNO155 (SHP2 inhibitor) + ribociclib as a phase I clinical trial (NCT04000529), and fourth line therapy with regorafenib were administered. No response was observed in all the above cases. Three months after regorafenib treatment, CT revealed that the GIST had grown to 18 cm, had invaded the transverse colon, and had increased the size of multiple liver metastases.

**Fig. 3:**
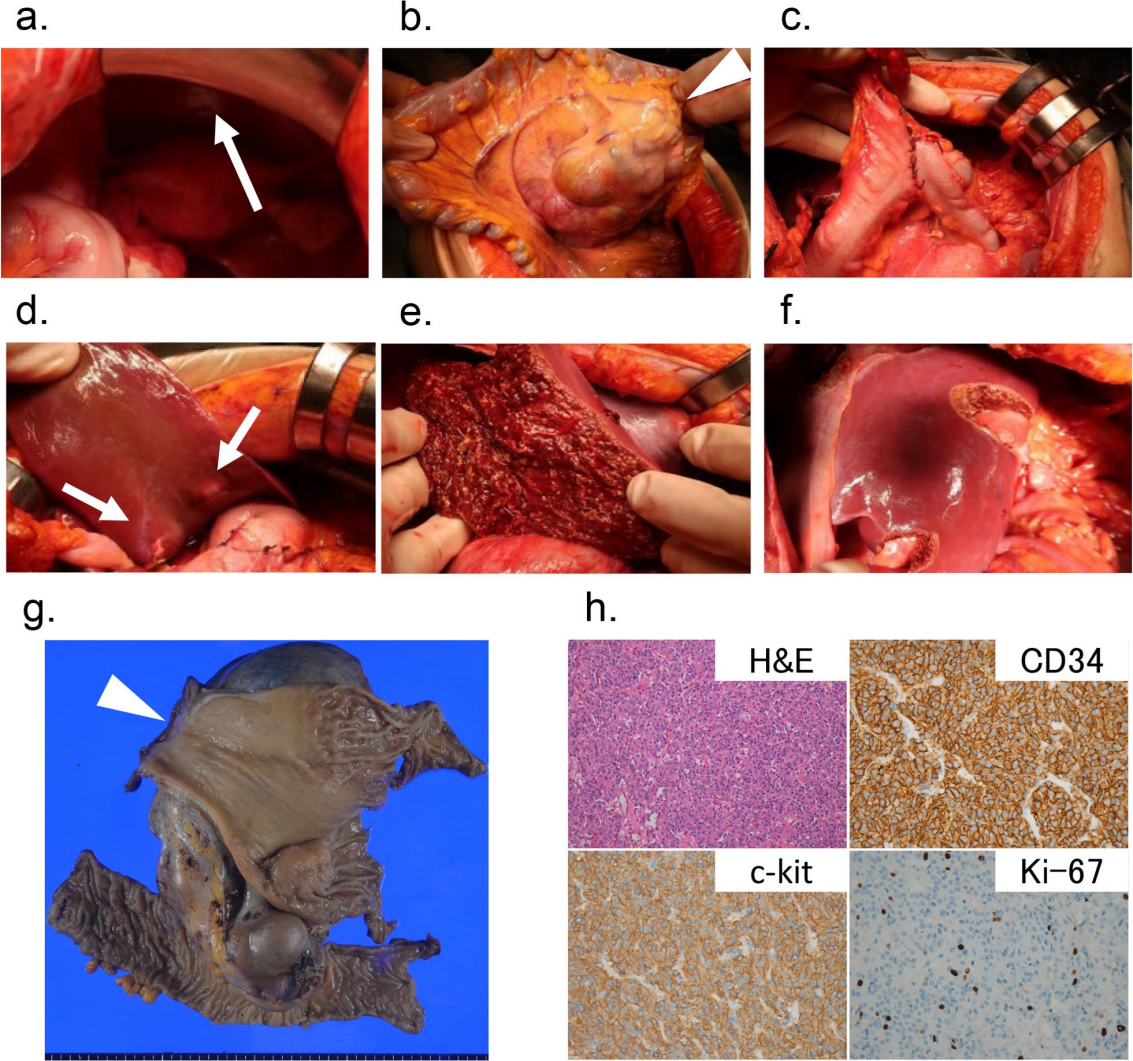
Intraoperative and pathological findings. **a** Incidental peritoneal metastases were confirmed in the pelvis (large arrow). **b** Huge gastric tumor invading transverse mesocolon is observed (arrowhead). **c** Distal gastrectomy and transverse colectomy were performed. **d** Multiple liver metastases were confirmed (small arrow). **e** Extended right posterior hepatic sectionectomy, including the S5 and S6 segments tumor was conducted. **f** Partial liver resection of the S1, S2, S3, S4, and S8 segments was performed. **g** Macroscopic appearance of the en bloc resected specimen showed that the mass, 18 × 11 cm in size, involving the transverse colon (arrowhead). **h** Hematoxylin–eosin staining of the resected tumor (× 20) revealed that tumor spindle cells were generally viable, and immunohistochemical staining demonstrated CD34 ( +), c-kit ( +), Ki-67 (+ 5%), indicating that the patient had a systemically drug-resistant GIST

The treatment strategy was carefully discussed again at the MDT conference, and a team of hepatobiliary-pancreatic surgeons was added to consider the necessity of resection of liver metastases and pancreatoduodenectomy for direct invasion of the pancreas and duodenum. Although best supportive care was also an option, the patient the patient’s age (20 years) and good general condition favored a more aggressive approach. Due to multidrug resistance, pharmacological therapy options were not expected to be effective. Surgical treatment to achieve R0 resection with curative intention or at least a prolonged prognosis with combination of debulking surgery and adjuvant drug therapy to prolong survival were the remaining options. After careful discussion, surgical resection by the hepatobiliary and pancreatic surgeons’ teams, including pancreatoduodenectomy or distal gastrectomy, transverse colectomy, and resection of liver metastases, was planned. The surgery was performed 23 months after the initial visit. On exploratory laparotomy, only three small nodules, in which peritoneal dissemination could not be ruled out, were detected in the pelvis (Fig. 3a). Although the tumor invaded the transverse colon, there was no pancreatic or duodenal invasion (Fig. 3b). Therefore, the tumor was resected. Initially, the peritoneal nodules were resected, and distal gastrectomy with Billroth I reconstruction and transverse colectomy were performed (Fig. 3c). Subsequently, partial liver resection (S1, S2, S3, S4, and S8 segments) and extended right posterior hepatic sectionectomy (including six metastatic tumors) were performed with the clamp crushing method using Pringle’s maneuver (Fig. 3d–f). The operative time was 6 h 3 min, and the estimated blood loss was 702 mL with no blood transfusion.

Macroscopic examination of the specimen revealed a whitish mass, 18 × 11 cm in size, involving the transverse colon, and 21 cm in diameter (Fig. 3g). Pathological examination confirmed that the tumor cells were generally viable. The immunohistochemical staining results were as follows: CD34 ( +), Ki-67 (+ 5%), and mitotic counts of < 5 per 50 high-power fields (Fig. 3h). Tumor presence was also confirmed in liver specimens and small nodules of the pelvis. The surgical margins were negative. The CD34 and c-kit positivity characterize GIST, whereas the Ki-67 index, although relatively low in our patient’s case, indicates proliferative activity. In addition, despite multiple lines of tyrosine and multi-kinase inhibitor therapies, the tumor cells remained viable, suggesting resistance to systemic drug treatment. Together these results indicate a drug-resistant disease. Genetic analysis revealed a succinate dehydrogenase (SDH) mutation, SDHA p.Y259fs*21. Therefore, this disease was likely associated with SDHA deficiency. No mutations were detected in KIT, platelet-derived growth factor receptor, or BRAF. Consequently, the final diagnosis was GIST with high-grade malignancy and moderate tumor progression risk according to Miettinen’s risk classification and high risk according to the Modified Fletcher Classification [8, 9], liver metastases, and peritoneal micro-metastases. According to Miettinen’s risk classification system, high-grade malignancies typically include tumors larger than 10 cm with a mitotic rate greater than 10 mitoses per 50 high-power fields, whereas our case showed a mitotic count of < 5 per 50 high-power fields, classifying it as moderate-risk according to this system.

The postoperative course was uneventful without complications, and the patient was discharged 14 days after surgery. Adjuvant imatinib therapy (400 mg/day) was administered and continued. After 27 months of follow-up, no clinical or radiological evidence of recurrence was observed.

## Discussion

The liver is the most frequent site of GIST metastasis, which poses a major threat to the survival of patients with GIST [10]. Although imatinib and other TKIs are standard treatments for patients with highly advanced and metastatic disease, surgical resection may be an optional treatment [11]. Several studies have examined the efficacy of surgical resection of advanced or metastatic GISTs, which may improve prognosis of these patients [12–19]. A Japanese prospective multicenter trial was conducted to clarify the efficacy and safety of surgery for liver oligometastasis in patients with GISTs. However, it was prematurely terminated due to guideline amendments and poor accrual [20]. Recently, Xue et al. reported the results of a single-institution study of 119 patients with liver metastases from GIST who were divided into hepatectomy and non-surgical groups. The results revealed that patients in the surgery group had significantly better progression-free survival (PFS) and marginally improved overall survival (OS) than those in the non-surgical group (3-year PFS: 86.2% vs. 64.6%, p = 0.002; 5-year OS: 91.5% vs. 78.3%, p = 0.083) [21].

The complete resection of residual metastatic GIST is a critical concern. Bauer et al. performed a multi-institutional analysis of PFS and OS in 239 patients with metastatic GIST who received imatinib therapy and underwent hepatectomy [22]. R0/R1 resection was achieved in 177 patients, and the median OS was 8.7 years in the R0/R1 patient group vs. 5.3 years in the R2 group (p = 0.0001). The median PFS among the R0/R1 group was not reached in the study and was 1.9 years in the R2 group. This suggests that R0 resection can improve the prognosis of patients with metastatic GIST. Although there is no known survival benefit from debulking surgery or R2 resection, there may be a possibility of improving resistance to imatinib and other TKIs through tumor reduction.

The clinical and biological features of GISTs in AYAs have been clarified. According to the United States National Cancer Institute, AYAs are defined as those aged 15–39 years at the time of initial cancer diagnosis. GISTs are uncommon among pediatric patients and AYAs, and the incidence rate of GIST in patients below 21 years of age ranges from 0.5 to 2.7% [10]. Some GISTs in children have been reported to be related to genetic syndromes, such as Carney–Stratakis syndrome [23]. Although most sporadic GISTs in AYAs are located in the stomach, heritable GISTs often occur in the small intestine [24]. Furthermore, the molecular characteristics of GISTs in AYAs are almost exclusively c-kit and platelet-derived growth factor receptor wild-type and mutations in the vast majority (85–90%) [25]. Regarding prognosis, a Dutch retrospective study demonstrated a 5-year survival of 85% in AYAs with GISTs, which has a relatively good prognosis compared with 76% in older adults [26].

However, reports focusing on GISTs with liver metastases in pediatric and AYA patients are limited. A systematic literature search was conducted using the PubMed database on May 17, 2024, to identify published reports on liver metastasis of GIST in children and AYAs. A combination of the following search terms was used: [(GIST [Title/Abstract] OR “gastrointestinal stromal tumor” [Title/Abstract]) AND (liver metastasis [Title/Abstract] OR hepatic metastasis [Title/Abstract])] AND (“Surgical Procedures, Operative” [MeSH Terms] OR "surgery" [Title/Abstract] OR “resection” [Title/Abstract]) AND (pediatric [Title/Abstract] OR adolescent [Title/Abstract] OR young adult [Title/Abstract] OR childhood [Title/Abstract] OR AYA [Title/Abstract]). This literature search revealed two case reports in the English-language medical literature, and the characteristics of these cases along with our case, are summarized in Table 1 [27, 28]. Li et al. reported on the case of a 12-year-old girl with GIST and liver metastases, highlighting the usefulness of fine-needle aspiration biopsy and resection of liver metastases in pediatric patients, with no recurrence at the time of reporting. Furthermore, Muniyappa et al. presented the case of a 16-year-old girl with gastric GIST and liver metastases, in which an open liver biopsy was performed simultaneously with resection of the primary gastric lesion, resulting in diffuse peritoneal metastasis 10 months after surgery. Although a good postoperative course was achieved with surgical resection in one case, a clear treatment strategy remains controversial. Surgical resection might be effective in cases of failure of fourth line treatment to prolong OS, improve the quality of life by relieving abdominal symptoms, and reduce drug resistance through tumor reduction, as long as the patient’s general condition is favorable and there is a possibility of achieving R0 resection. In the present case, the decision to perform surgery was determined by several factors: the young age of the patient, the ineffectiveness of previous treatment due to multidrug resistance, and the possibility that, even if R0 resection could not be achieved, a combination of debulking surgery and drug treatment might prolong the prognosis.

**Table 1:** Reported cases of GIST liver metastases in pediatric, adolescents and young adults populations undergoing surgery

References	Year	Age/Sex	Primary site	Primary tumor size (cm)	Metastatic site	Number of liver metastases	Preoperative drug therapy	Surgery	Genetic mutation	Postoperative drug therapy	Survival^*^ (month)
Li et al	2002	12/F	Stomach	13 × 4.5	Liver	2	None	1. Partial gastrectomy 2. Liver resection	None	N/D	N/D
Muniyappa et al	2007	16/F	Stomach	6.1 × 3.3 × 6.0	Liver	1	None	1. Partial gastrectomy 2. Open biopsy of the hepatic lesion	N/D	Imatinib	A (10)
Present case	2024	18/F	Stomach	18 × 11	Liver	11	1. Imatinib 2. Sunitinib 3. TNO155 + ribociclib 4. Regorafenib	1. Distal gastrectomy 2. Transverse colectomy 3. Liver resection	SDHA	Imatinib	A (27)

A, alive; GIST, gastrointestinal stromal tumors; N/D, no data; SDH, succinate dehydrogenase

^*^Survival time from surgery

Three years of adjuvant imatinib therapy improved recurrence-free survival (RFS) and OS in GIST patients with a high risk of GIST recurrence compared to 1-year of imatinib therapy (5-year RFS, 65.6% vs. 47.9%, p < 0.001; 5-year survival, 92.0% vs. 81.7%, p = 0.02) [29]. In the present case, the patient presented with microperitoneal dissemination and liver metastasis. Despite achieving R0 resection surgically, the tumor was deemed oncologically unresectable. Therefore, adjuvant imatinib therapy is planned to continue for more than 3 years.

## Conclusions

In conclusion, we experienced a successful R0 resection case of multidrug-resistant GIST invading the transverse colon with multiple liver metastases in a young woman. Even in highly advanced multidrug-resistant GISTs, surgery followed by adjuvant drug therapy may prolong the survival of young patients.

## Data Availability

The datasets analyzed in the present case report are not publicly available because of information that could compromise patient privacy. However, these are available from the corresponding author and can be obtained upon reasonable request.

## Abbreviations

AYA: Adolescent and young adult
CT: Computed tomography
FDG: 18F-Fluorodeoxyglucose
GISTs: Gastrointestinal stromal tumor
MDT: Multidisciplinary team
OS: Overall survival
PFS: Progression-free survival
RFS: Recurrence-free survival
SDH: Succinate dehydrogenase
SUVmax: Maximum standardized uptake value
TKIs: Tyrosine kinase inhibitor
